# Liposome Encapsulation of the Palmitoyl–KTTKS Peptide: Structural and Functional Characterization

**DOI:** 10.3390/pharmaceutics16020219

**Published:** 2024-02-02

**Authors:** Alberto Vitali, Patrizia Paolicelli, Barbara Bigi, Jordan Trilli, Laura Di Muzio, Vito Cosimo Carriero, Maria Antonietta Casadei, Stefania Petralito

**Affiliations:** 1 Istituto di Scienze e Tecnologie Chimiche “Giulio Natta”, National Research Council of Italy, Largo Francesco Vito 1, 00168 Rome, Italy; alberto.vitali@cnr.it; 2 Department of Drug Chemistry and Technologies, Sapienza University of Rome, Piazzale Aldo Moro 5, 00185 Rome, Italy; barbara.bigi@uniroma1.it (B.B.); jordan.trilli@uniroma1.it (J.T.); laura.dimuzio@uniroma1.it (L.D.M.); vitocosimo.carriero@uniroma1.it (V.C.C.); mariaantonietta.casadei@uniroma1.it (M.A.C.); stefania.petralito@uniroma1.it (S.P.)

**Keywords:** pal-KTTKS, liposomes, skin delivery, collagen production

## Abstract

In this study, the amphiphilic N-palmitoyl–KTTKS peptide was integrated in the bilayer of egg-derived phosphatidylcholine (PC) vesicles using two different preparation methods, namely thin-film evaporation (TLE) and reverse-phase evaporation (REV). Both the REV and TLE methods allowed for the formation of homogeneous liposome dispersions (PdI < 0.20) with mean hydrodynamic diameters of <100 nm and <200 nm, respectively, a net negative surface charge and a percentage of structured phospholipids higher than 90%. The inclusion of the amphiphilic N-palmitoyl–KTTKS peptide within phospholipid-based vesicles could improve peptide stability and skin delivery. Therefore, the obtained liposomes were evaluated via experiments assessing the synthesis of collagen and the ECM in 3T3-NIH fibroblasts. The obtained results showed that, when delivered with PC liposomes, pal-KTTKS stimulated collagen production more than free pentapeptide and 1 mM ascorbic acid, used as a positive control.

## 1. Introduction

Skin represents one of the most important organs of the human body, acting both as an extension and as the first natural physical barrier that protects the body from the external environment. Skin also has important physiological functions, such as the maintenance of the body’s water balance, the regulation of body temperature, and resistance to pathogenic invasion. Skin not only contributes toward maintaining internal homeostasis but also takes part in the metabolic processes of the human body. Thus, the preservation of skin integrity represents an important target in different fields, from regenerative medicine to cosmetics. Collagen is a major component of skin. It serves as the structural foundation of various tissues, including skin, bones, tendons, and cartilage, imparting strength, elasticity, and resilience. This multifaceted protein has garnered immense attention for its potential application in enhancing tissue regeneration and revitalizing the skin, playing a vital role in both regenerative medicine and cosmetics industry. Skin breakdown may derive from physiological aging or from pathologic-derived or external injuries. Skin aging is characterized by the weakening of the dermal collagen fiber network due to both decreased collagen production and deposition and increased proteolytic degradation, mainly by specific collagenases (MMPs) [1]. As a result, an overall impairment of all the extracellular matrix (ECM) occurs, thus reducing physiological and rheological skin properties and leading to an increased susceptibility to pathologies. To hinder this event, several mechanisms are put in place, including the production of specific molecules. In particular, many peptides are generated during skin damage, including matrikines or matricryptins [2], cryptic sequences hidden inside other proteins [3]. Matricryptins are generated throughout all phases of tissue healing and are actively involved in regulating inflammatory, fibrogenic, and angiogenic responses by binding to specific surface cellular receptors. Matrikines are peptides liberated by the partial proteolysis of ECM macromolecules, which are able to regulate cell activities. Among these peptides, some of them may modulate cell proliferation, cell migration, and protease production, which suggest that they can play a significant role in aiding the pathology of diseases and aging, as well as in tissue regeneration, as potential mechanisms of repair.

Matrikines or matricryptins have gained increasing attention in recent years for their ability to promote physiological remodeling and repair and also as topically applied biopeptides in cosmetic preparations. Bioactive peptides are increasingly emerging as molecules of choice to counteract pathological skin states because of their several advantages, such as their versatility, high target specificity, minimized toxicity and immunogenicity, and reduced costs of production thanks to innovative synthesis schemes [4]. The pentapeptide KTTKS (lysine–threonine–threonine–lysine–serine) is a matrikine originating from the proteolytic hydrolysis of collagen [5,6]. Besides its activity in promoting collagen production, this peptide has also been studied for its modulatory effect on cell migration [7,8]. The use of nanosystems for the delivery of these bioactive peptides is continuously increasing in order to preserve or enhance, and hence expand, their applicability [9]. However, there are several issues to consider when formulating peptides for topical delivery, from difficulties related to crossing the skin to the disadvantages of their high to very high molecular weight and polarity [10]. First, KTTKS must be absorbed into the stratum corneum. Once the compound has diffused through the stratum corneum, it must permeate through the epidermis, avoiding its proteolytic degradation. Once the KTTKS has reached and crossed the basement membrane, it will be able to exert its physiological effect. Since the stratum corneum is essentially lipophilic in nature, it provides the major rate-limiting barrier for KTTKS skin permeation. One of the strategies used to improve the topical delivery of KTTKS is to attach a lipophilic group (i.e., palmitic acid) to the N-terminal group of lysine through the formation of an amide bond, giving the N-palmitoyl–KTTKS derivative (pal-KTTKS), commercialized by Sederma Company in 2000 under the name Matrixyl and used in skin care products [11]. Among all fatty acids, palmitic acid has been chosen for KTTKS functionalization as it is one of the most represented in the stratum corneum; therefore, it may be expected to promote KTTKS penetration across the epidermal barrier. Indeed, a study by Choi et al., showed that the stability and permeability of pal-KTTKS was higher in epidermal and dermal skin extracts compared to KTTKS. Some investigations on the skin permeability of chemically modified peptides with palmitic acid have underlined that in the case of human epidermis, no detectable amounts of KTTKS and pal-KTTKS appear in the receptor phase after 48 h of exposure. According to the authors, since palmitic acid is a constituent of the intercellular lamellar lipid matrix of the human stratum corneum, and since both palmitic acid and KTTKS have linear structures, there is a possibility that pal-KTTKS remains trapped in the lamellar structure of the stratum corneum [12]. Therefore, to improve the skin permeability of pal-KTTKS, many nano-based drug delivery systems have been studied [13,14]. Particularly, lipid-based nanosystems such as liposomes, nanoemulsions, solid lipid nanoparticles, or nanostructured lipid carriers have been proposed for cutaneous application because they have been shown to enhance skin permeation and cellular uptake efficiency. Among these lipid-based delivery systems, liposomes are viewed as attractive delivery vehicles by those in the pharmaceutical industry due to their high biocompatibility, chemical composition variability, ease of preparation, and large variety of structural properties [15,16]. Liposomes are nanometric self-closed structures with a spherical shape and a diameter ranging from 20 nm upwards [17], capable of entrapping hydrophilic and hydrophobic drugs in their aqueous core and lipid bilayer, respectively, or even amphiphilic molecules, as is the case with pal-KTTKS. Embedding amphiphilic molecules within the double layer of liposome carriers produces a rearrangement of the phospholipid bilayer, leading to highly deformable vesicles. On the basis of these considerations, our strategy was to integrate the amphiphilic pal-KTTKS into the bilayer of egg-derived phosphatidylcholine (PC) vesicles, thus producing elastic nanocarriers able to enhance the skin delivery of the peptide. In particular, this encapsulation strategy should avoid the inconvenient aggregation of pal-KTTKS due to its amphiphilic nature, as observed during in vitro experiments [18,19]. Despite having a positive effect on human dermal fibroblasts, in more physiological conditions, such aggregation may produce a detrimental effect on pal-KTTKS bioavalilability. For this reason, pal-KTTKS was included in liposome vesicles which could protect it from aggregation. The obtained liposomes were investigated as potential enhancers for the skin delivery of pal-KTTKS; therefore, they were first evaluated in terms of vesicle size, zeta potential, and stability. Then, the delivery efficacy of the developed vesicles was evaluated by assessing the synthesis of collagen and the ECM in cultured 3T3-NIH fibroblasts.

## 2. Materials and Methods

### 2.1. Materials

Egg phosphatidylcholine (egg-PC) Lipoid 80 E was kindly offered by AVG srl (Bollate, MI, Italy). 4-(2-hydroxyethyl)-1-piperazine ethanesulfonic acid (HEPES), Bouin’s solution, and medium-grade Sephadex G-50 were purchased from Sigma-Aldrich (Saint Louis, MO, USA). Chloroform (CHCl_3_) was obtained from Merck (Kenilworth, NJ, USA). Diisopropyl ether, bidistilled water, sodium chloride (NaCl), ethanol, thiocyanatoiron, and 1,2-dichloroethane were supplied by CARLO ERBA Reagents (Cornaredo, MI, Italy). Cyclopore polycarbonate membrane filters (pore size 0.8, 0.4 and 0.2 µm, Whatman) were purchased from Merck (Kenilworth, NJ, USA). Pal-KTTKS (5P), at 96% purity, was purchased from Acadechem Company Ltd. (Kowloon, Hong Kong).

### 2.2. Liposome Preparation and Characterization

Plain liposomes (i.e., without pal-KTTKS) and liposomes with pal-KTTKS were prepared using two different techniques, thin layer evaporation (TLE) followed by extrusion and reverse-phase evaporation (REV) [20,21], in order to completely optimize the design of the nanocarriers. In detail, for the TLE procedure, 250 mg egg-PC were dissolved in a round-bottom flask containing 3 mL of chloroform. The organic solvent was evaporated using a rotary evaporator under reduced pressure until a thin lipid film was formed on the flask bottom. Any trace of organic solvent was further removed by connecting the flask to an oil rotary vane for 2 h. The dry lipid film was then hydrated with 5 mL of 1 mg/mL pal-KTTKS aqueous solution pH = 5 (the pH of the solution was adjusted with 0.1 N HCl). The obtained vesicles were downsized by sequential extrusion to form unilamellar liposomes. This step was performed through polycarbonate membrane filters of decreasing pore size (0.8, 0.4, and 0.2 μm) in order to obtain a narrow size distribution. More specifically, liposomes were sequentially extruded for 4 times through 0.8 μm membranes, 4 times through 0.4 μm membranes, and 8 times through 0.2 μm membranes. To remove the unencapsulated pal-KTTKS, the sample was subjected to a gravity flow size exclusion chromatography (SEC) column packed with Sephadex G-50 resin. The pal-KTTKS-loaded liposomes obtained with the TLE procedure were named TLE5P. The same procedure was used to prepare plain liposomes (i.e., liposomes not containing pal-KTTKS). This sample was named TLE. For the REV procedure, liposomes were prepared as described by Szoka et al. [22]. Briefly, 50 mg of egg-PC was dispersed in 3 mL of chloroform/diisopropyl ether 1:1 *v*/*v* mixture (supplemented with 1 mg of pal-KTTKS for REV5P samples). Successively, the solution was mixed with 1 mL of HEPES buffer (10 mM, pH = 7.4), and the mixture was sonicated in an ultrasonic bath (40 kHz, Argo Lab Digital Ultrasonic Cleaner DU65), above the lipid main transition temperature (Tm), for 20 min to form a stable emulsion. The organic solvents were removed using a rotary evaporator until a viscous gel was formed. The volume was adjusted to 2 mL by the addition of 10 mM HEPES buffer (pH = 7.4). At the end of the procedure, to eliminate any traces of the organic solvents, the liposomal sample was further subjected to slow evaporation using a rotary evaporator and a subsequent flow of gaseous nitrogen. The unencapsulated pal-KTTKS was then removed using a gravity flow SEC column packed with Sephadex G-50 resin. The plain and pal-KTTKS-loaded liposomes obtained with the REV procedure were named REV and REV5P, respectively.

### 2.3. Physicochemical Characterization of Liposomes

After the preparation of the liposomes, the physico-chemical characterization of all the formulations was carried out based on the quantitative determination of structured phospholipids by the Yoshida assay and size analysis.

#### 2.3.1. Determination of Hydrodynamic Diameter and Size Distribution

Size analysis (hydrodynamic diameter and size distribution) was performed using a Zetasizer (Malvern Instruments Ltd., Malvern, UK) at a constant temperature of 25.0 ± 0.1 °C. All the analyses were carried out in triplicate, and the mean, standard deviation, and relative standard deviation values were calculated.

#### 2.3.2. Determination of ζ-Potential

The ζ-potential of the liposome formulations was measured using particle microelectrophoresis (Zetasizer Nano ZS-90, Malvern Instruments, Worcestershire, UK). For zeta potential analysis, the samples were diluted 1:100 with 10 mM HEPES buffer (pH = 7.4) prior to measurements in order to avoid multiple scattering effects.

#### 2.3.3. Phospholipid Assay

The phospholipid concentration was determined with the Yoshida assay [23], one of the traditional methods used after liposome preparation. This assay is based on the ability of phospholipids to form a colored complex with ammonium ferrothiocyanate, whose light absorption is converted into a weight amount of phospholipids. An aliquot of the liposomal samples (pre-SEC and post-SEC) equal to 0.4 mL is taken; then, 0.2 mL of ethanol, 1 mL of ferric thiocyanate solution (previously prepared by solubilizing 0.97 g of nitrate ferric acid and 15.2 g of ammonium thiocyanate in 100 mL of distilled water), 0.6 mL of 0.17 N HCl, and 3 mL of 1,2-dichloroethane are added. The test tube was vortexed for 2 min using a ZX3 vortex mixer and subsequently centrifuged at 2000 rpm for 5 min in order to achieve phase separation. The aqueous phase was discarded, whereas the absorbance of the organic phase, containing the hydrophobic ferric thiocyanate–phospholipid complex, was analyzed at λ = 480 nm using the Perkin Elmer UV/Vis Lambda 25 spectrophotometer. The percentage of structured lipids was determined using a suitable calibration line that was previously constructed using several known lipid concentrations. All the analyses were carried out in triplicate, and the mean, standard deviation, and relative standard deviation values were calculated.

#### 2.3.4. Effect of Culture Medium on Vesicle Stability

The samples prepared using the TLE and REV procedures were diluted 1:100 in 10 mM HEPES (pH = 7.4) or DMEM culture medium in order to evaluate their colloidal stability over time. The samples were stored at 37 °C, and after 24 and 48 h, they were analyzed for size and size distribution.

### 2.4. Cell Culture and Cytotoxicity Assays

Murine fibroblasts 3T3-NIH were cultured in DMEM supplemented with 10% FBS and penicillin (100 units/mL)–streptomycin (100 units/mL) in a humidified atmosphere with 5% CO_2_ (37 °C). The cells (8000 cells/well) were seeded into 96-multiwell plates containing DMEM (100 μL) and incubated for 24 h to allow the cells to adhere. Once the cells had reached 80–90% confluence, they were transferred to the 96-well plates before sample inoculation was performed. The samples were represented by free pal-KTTKS (concentration 5 μM, 10 μM and 20 μM), plain liposomes (TLE sample), and pal-KTTKS-loaded liposomes (TLE5P sample) at lipid concentrations of 10 μg/mL, 100 μg/mL, 200 μg/mL, and 400 μg/mL, corresponding to pal-KTTKS concentrations of 0.25 μM, 2.5 μM, 5 μM, and 10 μM. After 24 and 48 h of treatment, a Cell-Titer Blue Assay (Promega, Madison, WI, USA) was carried out according to the manufacturer’s instructions to analyze cell viability, expressed as a percentage, versus non-treated cells. During the incubation period (1–4 h at 37 °C), the resulting fluorescence of the dye was determined every hour until signal stabilization using an automatic microplate reader (GloMax Multi Detection Microplate Reader, Promega, Madison, WI, USA) using a 525 ex/580 em–640 filter. Cell viability, expressed as a percentage, was evaluated considering control cells at 100%. To determine the cell viability percentage, Equation (1) was employed:

(1) $$\mathrm{Cell}\;\mathrm{viability}\;\left(\%\right)=\frac{\mathrm{fluorescence}\;\mathrm{of}\;\mathrm{the}\;\mathrm{treated}\;\mathrm{sample}}{\mathrm{fluorescence}\;\mathrm{of}\;\mathrm{the}\;\mathrm{control}\;\mathrm{sample}}\times 100$$

### 2.5. Sirius Red Assay for Collagen Detection

This assay was carried out on a murine fibroblast 3T3-NIH cell line, the cells of which, once 80–90% confluence was reached, were transferred onto a 24-well plate (culture surface 1.9 cm^2^; working volume 1 mL). The cells were treated with TLE5P and REV5P at lipid concentrations 200 μg/mL and 400 μg/mL (corresponding to peptide concentrations of 5 μM and 10 μM, respectively). Plain TLE and REV samples at the same lipid concentrations, and free pal-KTTKS at concentrations of 5 μM and 10 μM, were used as controls. In addition, a negative control (PBS) and a positive control, represented by 1 mM ascorbic acid, were used. Before sample inoculation, they were sterilized by UV exposure for ten minutes. The samples were inoculated in duplicate for each assay. After 72 h, the removal of the medium and subsequent washings with PBS took place. At this point, to fix the cells in situ, 70% (*v*/*v*) ethanol was added to each well, and we ensured we covered the entire surface, and the plates were subsequently stored at −80 °C for ten minutes. After this time, the hydroalcoholic solution was removed and treatment with Sirius Red dye was performed. Such a dye is capable of selectively staining Type I and Type III collagen. This occurs because, in an acidic environment, Sirius Red goes on to interact with the basic groups of collagen, staining it red [24]. Briefly, the cells were fixed by adding 300 μL Bouin’s solution in each well for 1 h at r.t. Bouin’s solution was removed, and the plates were subsequently rinsed with cold water via a pipette until the yellowish solution due to the staining was no longer visible. The plates were then allowed to dry in a fume hood overnight. Next, 200 μL of Sirius Red dye solution (50 mg Sirius Red F3BA dissolved in 50 mL of water-saturated picric acid) was added to each well and left to stay for 1 h at r.t. At the end of the treatment, the unbound dye was removed, and two washes with distilled water were carried out, followed by a final one with 0.01 M HCl. A visual analysis was carried out under light microscope, and images of all wells were taken using a professional camera (Nikon D3100 mounted on microscope oculars). Thereafter, the cells were washed with a NaOH 1M solution to recover the bound stain, which was evaluated using a ELx800 microplate reader (Biotek, Rodano (MI), Italy) with a 560 nm filter. The results were reported considering a control percentage of 100%.

### 2.6. Statistical Analysis

For our in vitro cytotoxicity and collagen production assays, analyses were carried out to evaluate the effects of the treated vs. control cell samples. Data were obtained via triplicate experiments, and the significance levels are reported in the legends of figures. The statistical significance of the data was determined using GraphPad Prism version 8 (GraphPad Software, San Diego, CA, USA) via a one-way analysis of variance at *p* < 0.05 (ordinary one-way ANOVA) and performing multiple comparisons with the control.

## 3. Results and Discussion

### 3.1. Liposome Preparation and Characterization

Two different techniques, TLE and REV, were used for the preparation of pal-KTTKS-loaded liposomes. Initial attempts were made to obtain pal-KTTKS-loaded liposomes through TLE dissolving pal-KTTKS in the organic solvent together with egg-PC. However, it was not possible to obtain a homogeneous lipid film in this case. Therefore, pal-KTTKS was included in the aqueous solution used to hydrate the dry lipid film. To do that, it was necessary to dissolve pal-KTTKS in slightly acidic aqueous solution, because pal-KTTKS assemble into micelles when dissolved in 10 mM HEPES buffer (pH = 7.4) at a concentration of 1 mg/mL. Such conditions were chosen because it is better to have amphiphilic molecules like pal-KTTKS in their free monomeric form during liposome preparation in order to promote the integration of the palmitoyl chain within the phospholipid bilayer. For the same reason, a different preparation technique, REV, was also used to obtain pal-KTTKS-loaded liposomes. Indeed, the REV method allowed for the dissolving of both pal-KTTKS and egg-PC in the organic phase prior to emulsification with 10 mM HEPES buffer (pH = 7.4). Such experimental conditions could promote the integration of the palmitoyl chain of pal-KTTKS within the phospholipid bilayer. Moreover, the REV technique allowed for us to obtain liposomes dispersed in a more physiological medium compared to TLE.

Dynamic light scattering and zeta potential measurements were used to evaluate the effect of pal-KTTKS on the particle size and surface charge of liposomes prepared by the two different procedures, namely TLE and REV. The results obtained for samples loaded with pal-KTTKS (labeled TLE5P and REV5P) are reported in Table 1 and compared with those for plain liposomes (labeled TLE and REV samples).

The obtained results highlight that both the REV or TLE methods are suitable, with their utilization leading to percentages of structured phospholipids in liposomes higher than 90% while forming homogenous populations of liposomes with a size < 100 nm (REV method) or <200 nm (TLE method) and PdI < 0.20.

No significant interference with vesicle formation was observed with pal-KTTKS, as suggested by the percentages of phospholipid-forming vesicles, which are very similar for plain (TLE and REV) and pal-KTTKS-loaded liposomes (TLE5P and REV5P), irrespective of the type of procedure used. In fact, the percentage of lipid molecules recovered from the loaded liposomes only partially decreased compared to plain vesicles, suggesting that the hydration step of liposome preparation was only slightly influenced by the presence of pal-KTTKS.

The size range of the samples was considered appropriate for transdermal delivery [25]. It was previously reported that only particles with a mean size ranging from 50 to 500 nm are able to penetrate into the skin [26]. The slight average size increase observed for the 5P samples (TLE5P and REV5P) compared to the plain samples (TLE and REV) is consistent with the deformation of the bilayer, which, being in the immediate vicinity of pal-KTTKS, could wrap around it.

**Figure 1:**
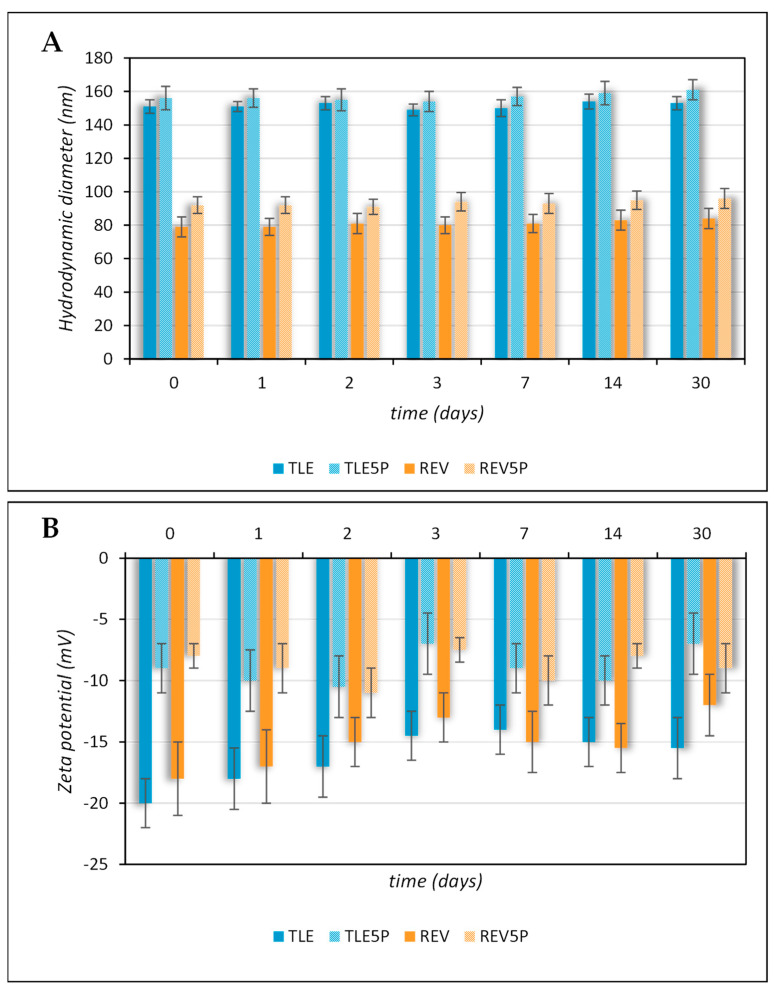
Colloidal stability of plain and pal-KTTKS-loaded liposomes obtained by TLE and REV and stored at 4 °C. (**A**) Hydrodynamic diameter; (**B**) zeta potential.

The zeta potential values of the plain liposomes prepared by TLE or REV are comparable. The less negative zeta potential values of the REV5P and TLE5P formulations are due to a change in the net negative surface charge in the presence of pal-KTTKS. This result is in agreement with the chemical nature of the loaded material. In fact, pal-KTTKS is characterized by ionizable amino acid residues which are positively charged below their pKa at pH = 7.4. (the samples for the measurement of zeta potential were diluted in HEPES buffer at pH = 7.4). The measurement of zeta potential therefore allows us to confirm the presence of the peptide on the surface of the liposomal vesicles. The pal-KTTKS decreases the zeta potential value of the vesicles, possibly leading to an increase in the chemical–physical instability of the samples containing it. Therefore, it was necessary to monitor the stability of these samples with respect to the corresponding plain formulations. Furthermore, it must be considered that the pal-KTTKS could also act as an agent that destabilizes the lipid arrangement due to its amphiphilic nature and surfactant-like properties. As it is known, lipid bilayer membranes are mechanically destabilized by the presence of amphiphilic compounds like surfactants, causing the breakdown of the bilayer. For this reason, changes in size over time were evaluated in all tested samples. All samples remained stable with respect to size, size distribution, and zeta potential for up to 30 days, as can be observed in Figure 1A,B. Furthermore, they remained stable over 48 h at 37 °C in DMEM culture medium. As can be seen from the average hydrodynamic diameter and PdI values reported in Table 2, neither a temperature of 37 °C nor the culture medium caused instability in the systems investigated.

### 3.2. Cytotoxicity Assay

**Figure 2:**
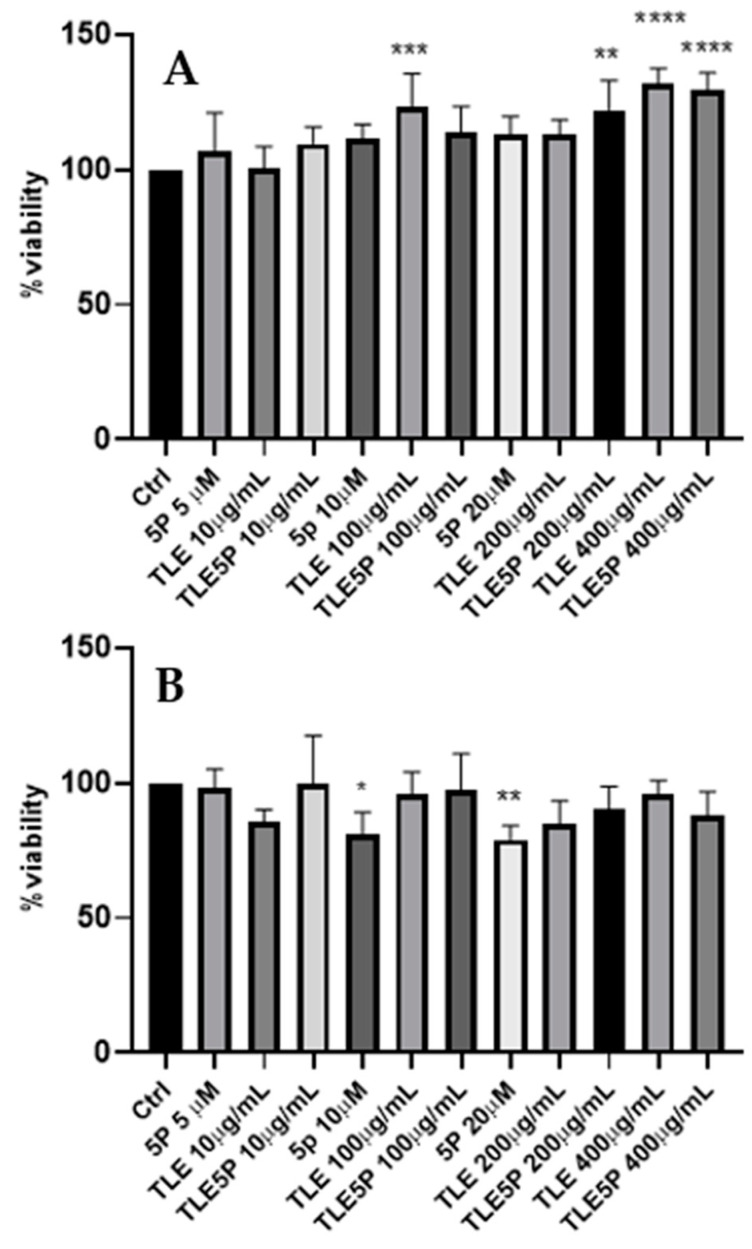
MTS cytotoxicity assay. Ctrl: control. For the other acronyms, refer to the Section 2. (**A**): 3T3-NIH cells after 24 h of treatment. (**B**): cells after 48 h of treatment. All data are represented as the mean (SD) (* *p* < 0.05, ** *p* < 0.01, *** *p* < 0.001, **** *p* < 0.0001).

In order to evaluate the potential cytotoxic effect of the various preparations, experiments that involved exposing the fibroblast 3T3-NIH cell line to different concentrations of liposome preparations were carried out. The results reported in Figure 2 were obtained from our MTS test analysis. Treatments were compared to cells treated only with PBS used as vehicles of all the preparations vehicles (negative control). Afterward, plain liposomes, free pal-KTTKS, and pal-KTTKS-loaded liposomes were added to each well and incubated for 24 h and 48 h at various final concentrations. The concentrations of pal-KTTKS used in this test were chosen according to other studies [6] and to cover concentrations also above those used in this study with the encapsulated peptide. As a result, all the used preparations were not cytotoxic to 3T3-NIH fibroblasts, neither at 24 h nor at 48 h, as the minimal viability was recorded to be 78% for the 48 h treatment with pal-KTTKS at 20 μM (Figure 2B), less than 70%.

The lack of cytotoxicity in these formulations is of crucial importance for potential applications in cosmetic or even regenerative medicine fields.

#### Collagen Production upon Liposome Treatment

**Figure 3:**
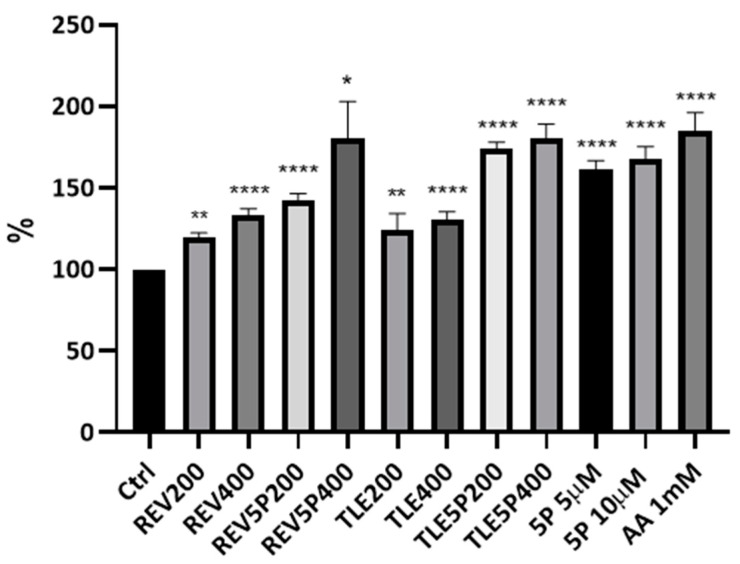
Collagen production by NIH-3T3 fibroblasts upon treatment with liposomes and loaded-liposome preparations. The Sirius Red colorimetric assay was carried out using a microplate reader with filters set at 560 nm. Results are percentages with respect to the Ctrl (control) (considered as 100%). For the other acronyms, refer to the Material and Methods section. AA: ascorbic acid; 5P: palmitoyl-pentapeptide. All data are represented as the mean (SD) (* *p* < 0.05, ** *p* < 0.01, **** *p* < 0.0001).

**Figure 4:**
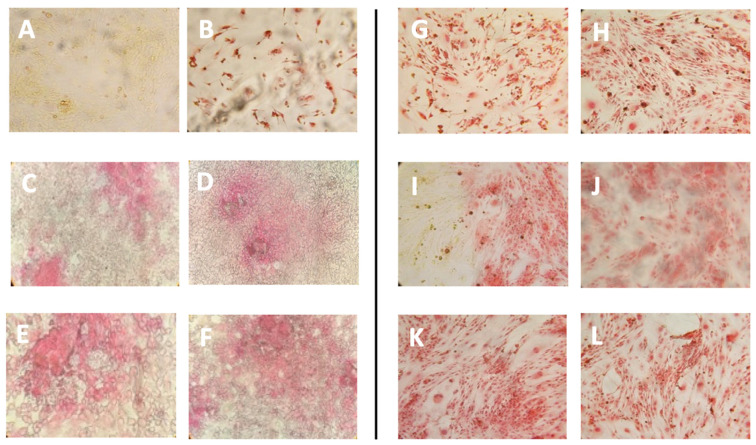
Phase-contrast images of Sirius Red staining of NIH-3T3 fibroblasts upon treatment with the different liposome preparations. (**A**): control, untreated cells; (**B**): cells treated with ascorbic acid 1 mM; (**C**): cells treated with REV200 liposomes (**D**): REV400; (**E**): TLE 200; (**F**): TLE 400; (**G**): cells treated with pal-KTTKS 5 μM; (**H**): cells treated with pal-KTTKS 10 μM; (**I**): REV5P 200; (**J**): REV5P 400; (**K**): TLE5P 200; (**L**): TLE5P 400. REV: liposomes obtained through the reverse-phase evaporation method; TLE: liposomes obtained through the thin-layer evaporation method. REV 200 and TLE 200: plain liposomes tested at a lipid concentration of 200 µg/mL; REV 400 and TLE 400: plain liposomes tested at a lipid concentration of 400 µg/mL. REV5P 200 and TLE5P 200: pal-KTTKS-loaded liposomes tested at a lipid concentration of 200 µg/mL; REV5P 400 and TLE5P 400: pal-KTTKS-loaded liposomes tested at a lipid concentration of 400 µg/mL).

pal-KTTKS is a well established agent that promotes collagen production by fibroblasts and keratinocytes cells [6,18,27]. To establish whether the liposome preparations were able to retain this activity, a Picro-Sirius Red colorimetric assay was set up on NIH-3T3 fibroblasts. The Sirius Red assay is a colorimetric method that allows for the qualitative and quantitative analysis of collagen synthesis both in tissues, and thus in histochemical analysis, and in cell cultures [24,28]. The peptide concentrations tested were 5 μM and 10 μM, whereas the lipid concentrations of the liposomal formulations delivering this pentapeptide concentration (TLE5P and REV5P) were 200 μg/mL (corresponding to a peptide concentration of 5 μM) and 400 μg/mL (corresponding to a peptide concentration of 10 μM). Cells were treated with free (5P) and encapsulated peptide (REV5P and TLE5P). In addition, both a negative control (PBS buffer) and a positive control, represented by 1 mM ascorbic acid, were used; ascorbic acid at this concentration is commonly used to stimulate collagen production in several cell types [29,30]. As expected, the positive control and the free form of pal-KTTKS, at 5 and 10 μΜ, produced a positive effect on the production of collagen. When included in liposomes, the pal-KTTKS was still able to stimulate collagen production with respect to the control, as shown by the results derived from the Sirius Red staining assay (Figure 3). In particular, a higher rate of collagen production was observed for the REV5P and TLE5P liposome preparations when tested at a lipid concentration of 400 μg/mL, values that are comparable to the ascorbic acid treatment but indicate greater effectiveness compared to the free pal-KTTKS (Figure 3). A similar trend was observed in the phase-contrast microscopy images of cells stained with Sirius Red (Figure 4), where the deposition of the red stain indicating the presence of collagen within cells was produced. The intensity of the stain is evident in the positive controls (ascorbic acid 1 mM and free pal-KTTKS, Figure 4B,G and H, respectively) with a pattern of coloration similar to that observed by other groups in similar experiments [18,24] and in liposome-encapsulated peptide treatments (Figure 4C–F,I–L). A similar result regarding collagen production induced by pal-KTTKS conjugated with ionic liquids was observed by Gomes et al. [31].

The major effectiveness of pal-KTTKS encapsulated in liposomes with respect to the free form may be due to two different effects. On the one hand, the encapsulation avoids the phenomenon of the aggregation and microneedle formation of pentapeptides in aqueous solution, as observed elsewhere [18]. This event was also observed during our experiments, and surely this effect may reduce the bioavailability of the pal-KTTKS. On the other hand, an enhanced interaction with cell membrane structure is promoted by liposomes favoring the biological effect of the peptide. A strategy to encapsulate lipopeptides in liposome vesicles has been successfully adopted in other studies for the delivery of vaccines [32] or for the transport of drugs through the Brain–Blood Barrier [33]. Interestingly, as can be observed in Figure 3 and Figure 4, plain liposomes (TLE and REV samples) were also shown to be able to induce collagen production, although with a less potent effect compared to the pal-KTTKS-loaded liposomes. In this sense, amongst the unloaded liposome preparations, TLE 200 and TLE 400 were found to be the most effective.

From the results obtained in this study, it can be stated that the liposome formulation maintains the properties of the free peptide while adding some advantages. Liposomes can improve the penetration of pal-KTTKS into the skin. It is known that the skin permeability of both KTTKS and pal-KTTKS, when used in their free forms, is low [11]; thus, liposome formulations such as those proposed in this study may greatly improve the applicability of pal-KTTKS, avoiding the use of invasive techniques such as those involving microneedle technology [34]. Furthermore, liposomes provide a protective environment for the encapsulated peptide, helping to stabilize it, enabling a controlled and sustained release to prolong the presence of the active ingredient in the skin, potentially leading to longer-lasting anti-aging effects. Liposomal delivery can ultimately enhance the bioavailability of pal-KTTKS, meaning that a greater proportion of the active ingredient reaches its target within the skin, improving the overall efficacy of the product.

## 4. Conclusions

Given the interesting features of the pentapeptide derivative palmitoyl–KTTKS, in this study, we evaluated the possibility of encapsulating it in liposome nanosystems in order to ameliorate its bioavailability, as KTTKS and pal-KTTKS have been shown to have null skin permeability and limited skin permeability, respectively [12]. Another crucial aspect that may improve this peptide’s efficacy, deriving from its liposome encapsulation, is its possible resistance to proteolytic degradation due to epidermal proteolytic enzymes such as aminopeptidesases. The encapsulation scheme we obtained in this study provides a new efficient formulation of pal-KTTKS in terms of collagen production, showing that the encapsulated peptide not only maintains the original activity but also ameliorates it. This effect could potentially expand the applications of this bioactive peptide to fields other than cosmetics, hinting at the potential for liposomes to be administered through parenteral, oral, pulmonary, nasal, ocular, and transdermal routes [9], with successful instances of the latter being reported in [35]. The enhanced production of collagen by liposome-encapsulated palmitoyl–KTTKS versus a free peptide can also significantly impact wound healing processes. Harnessing this capability may lead to the development of advanced wound care therapies and formulations that facilitate quicker tissue repair, reduce scarring, and enhance overall skin regeneration. To achieve these aims, further studies aiming to monitor the permeability of this nanoformulation in appropriate skin models are needed.

## Figures and Tables

**Table 1:** Characterization results based on the following: ^a^ intensity size distribution model, ^b^ polydispersity index, ^c^ zeta potential in HEPES buffer at pH = 7.4, and ^d^ % of structured phospholipids in the liposomes (as determined by the Yoshida assay).

Sample	Hydrodynamic Diameter ^a^ Mean (SD)	PdI ^b^ Mean (SD)	Zeta Potential ^c^ Mean (SD)	% of Structured Phospholipid ^d^
TLE	151 (4) nm	0.11 (0.08)	−20.58 (1.85) mV	94
REV	80 (6) nm	0.19 (0.04)	−18.58 (3.00) mV	95
TLE5P	156 (7) nm	0.11 (0.02)	−9.08 (0.65) mV	92
REV5P	92 (5) nm	0.19 (0.01)	−8.58 (0.65) mV	91

**Table 2:** Stability of plain and pal-KTTKS-loaded liposomes obtained by TLE and REV. The samples were diluted in HEPES buffer (pH = 7.4) and in DMEM culture medium.

Sample		t0		t24 h		t48 h	
		HEPES	DMEM	HEPES	DMEM	HEPES	DMEM
TLE	Hydrodynamic Diameter (nm)	157	138	155	144	148	142
	PdI	0.09	0.30	0.08	0.20	0.09	0.2
TLE5P	Hydrodynamic Diameter (nm)	182	134	161	143	169	144
	PdI	0.20	0.20	0.20	0.20	0.20	0.10
REV	Hydrodynamic Diameter (nm)	86	79	89	126	88	137
	PdI	0.20	0.30	0.20	0.20	0.20	0.20
REV5P	Hydrodynamic Diameter (nm)	84	73	83	106	86	111
	PdI	0.20	0.30	0.20	0.20	0.20	0.20

## Data Availability

All generated data are included in this article.
